# 2-(2-Nitro­phen­yl)-1,3-benzothia­zole

**DOI:** 10.1107/S1600536812029844

**Published:** 2012-07-07

**Authors:** S. Vijayakumar, S. Murugavel, R. Selvakumar, M. Bakthadoss

**Affiliations:** a Department of Physics, Sri Balaji Chokkalingam Engineering College, Arni, Thiruvannamalai 632 317, India; b Department of Physics, Thanthai Periyar Government Institute of Technology, Vellore 632 002, India; c Department of Organic Chemistry, University of Madras, Maraimalai Campus, Chennai 600 025, India

## Abstract

In the title compound, C_13_H_8_N_2_O_2_S, the essentially planar benzothia­zole system [maximum deviation = −0.012 (1) Å for the S atom] is oriented at a dihedral angle of 48.3 (1)° with respect to the benzene ring. The nitro group is substanti­ally twisted from the plane of its attached benzene ring [dihedral angle = 52.0 (1)°]. The crystal packing features C—H⋯O hydrogen bonds, which generate *C*(6) helical chains propagating along [010]. Weak C—H⋯π inter­actions also occur in the crystal.

## Related literature

For the pharmacological activity of benzothia­zole derivatives, see: Repiĉ *et al.* (2001 ▶); Schwartz *et al.* (1992 ▶). For related structures, see: Lakshmanan *et al.* (2011 ▶); Zhang *et al.* (2008 ▶).
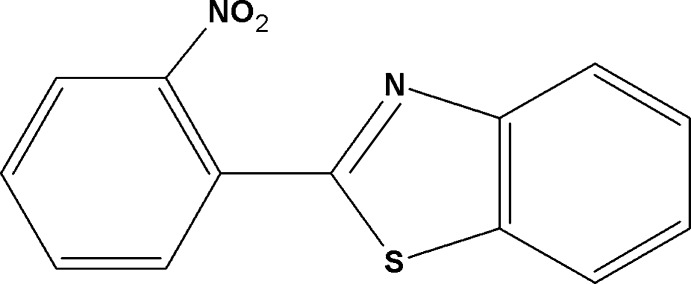


## Experimental

### 

#### Crystal data

C_13_H_8_N_2_O_2_S
*M* *_r_* = 256.27
Monoclinic, 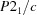

*a* = 7.6092 (2) Å
*b* = 12.7854 (3) Å
*c* = 11.9938 (3) Å
β = 90.556 (2)°
*V* = 1166.78 (5) Å^3^
*Z* = 4
Mo *K*α radiation
μ = 0.27 mm^−1^
*T* = 293 K
0.24 × 0.22 × 0.16 mm

#### Data collection

Bruker APEXII CCD diffractometer
Absorption correction: multi-scan (*SADABS*; Sheldrick, 1996 ▶) *T*_min_ = 0.937, *T*_max_ = 0.958
14037 measured reflections
3258 independent reflections
2559 reflections with *I* > 2σ(*I*)
*R*_int_ = 0.027

#### Refinement

*R*[*F* ^2^ > 2σ(*F* ^2^)] = 0.039
*wR*(*F* ^2^) = 0.113
*S* = 1.05
3258 reflections
163 parameters
H-atom parameters constrained
Δρ_max_ = 0.24 e Å^−3^
Δρ_min_ = −0.32 e Å^−3^

### 

Data collection: *APEX2* (Bruker, 2004 ▶); cell refinement: *APEX2* and *SAINT* (Bruker, 2004 ▶); data reduction: *SAINT* and *XPREP* (Bruker, 2004 ▶); program(s) used to solve structure: *SHELXS97* (Sheldrick, 2008 ▶); program(s) used to refine structure: *SHELXL97* (Sheldrick, 2008 ▶); molecular graphics: *ORTEP-3* (Farrugia (1997 ▶); software used to prepare material for publication: *SHELXL97* and *PLATON* (Spek, 2009 ▶).

## Supplementary Material

Crystal structure: contains datablock(s) global, I. DOI: 10.1107/S1600536812029844/hb6879sup1.cif

Structure factors: contains datablock(s) I. DOI: 10.1107/S1600536812029844/hb6879Isup2.hkl

Supplementary material file. DOI: 10.1107/S1600536812029844/hb6879Isup3.cml

Additional supplementary materials:  crystallographic information; 3D view; checkCIF report

## Figures and Tables

**Table 1:** Hydrogen-bond geometry (Å, °) *Cg*1, *Cg*2 and *Cg*3 are the centroids of the S1/N1/C1/C2/C7 thiazole ring, the C2–C7 benzene ring and the C8–C13 benzene ring, respectively.

*D*—H⋯*A*	*D*—H	H⋯*A*	*D*⋯*A*	*D*—H⋯*A*
C11—H11⋯O1^i^	0.93	2.51	3.236 (2)	135
C9—H9⋯*Cg*1^ii^	0.93	2.92	3.468 (2)	119
C10—H10⋯*Cg*2^ii^	0.93	2.90	3.536 (2)	127
C3—H3⋯*Cg*3^iii^	0.93	2.99	3.673 (2)	132

Symmetry codes: (i) 
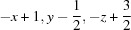
; (ii) 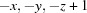; (iii) 
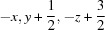
.

**Fig. 1.**
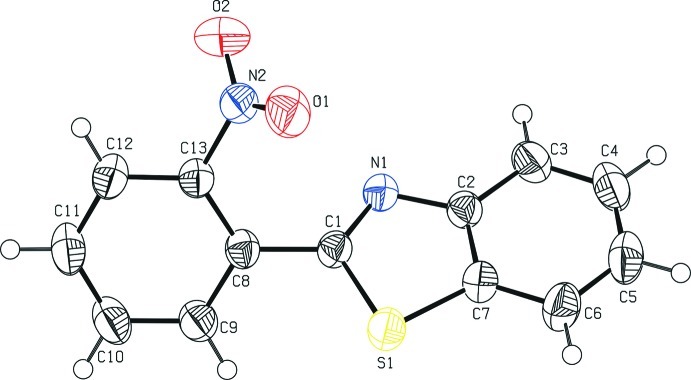
The molecular structure of the title compound. Displacement ellipsoids are drawn at the 50% probability level.

**Fig. 2.**
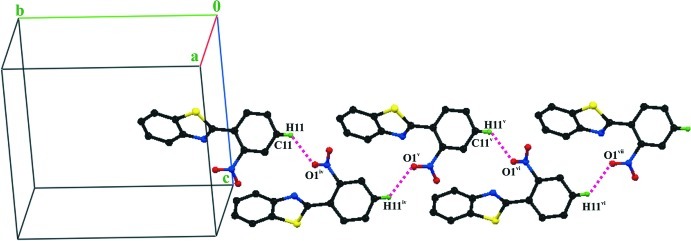
Part of the crystal structure of (I) showing intermolecular C—H···O hydrogen bonds (dotted lines), forming C(6) zigzag chains along the b axis. For clarity H atoms involved in the hydrogen bonds are shown. [Symmetry codes:(iv)1 - x, -1/2 + y, 3/2 - z; (v)x, -1 + y, z; (vi)1 - x, -3/2 + y, 3/2 - z; (vii)x, -2 + y, z].

**Fig. 3.**
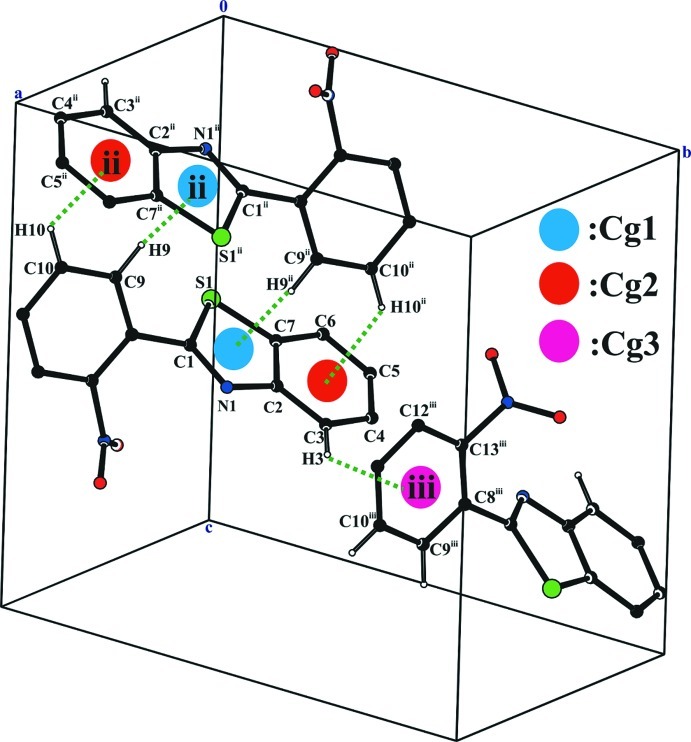
A view of the C—H···π interactions (dotted lines) in the crystal structure of the title compound. Cg1, Cg2 and Cg3 denotes centroid of the S1/N1/C1/C2/C7 thiazole ring, C2–C7 benzene ring and C8–C13 benzene ring, respectively. [Symmetry codes: (ii)-x, -y, 1 - z; (iii)-x, 1/2 + y, 3/2 - z].

## supplementary crystallographic information

### Comment

The benzothiazole nucleus is associated with several pharmacological activities such as anti-tumor (Repiĉ *et al.*, 2001) and antimicrobial (Schwartz *et al.*, 1992). As part of our studies in this area, the crystal structure of the title compound has been determined and the results are presented here.

Fig. 1. shows a displacement ellipsoid plot of (I), with the atom numbering scheme. The benzothiazole moiety (S1/N1/C1—C7) is essentially planar [maximum deviation = -0.012 (1) Å for the S atom] and lies at an angle 48.3 (1)° with respect to the benzene ring. The nitro group (N2/O1/O2) is twisted from the attached benzene ring, forming a dihedral angle of 52.0 (1)°. The geometric parameters of the title molecule agrees well with those reported for similar structures (Lakshmanan *et al.*, 2011, Zhang *et al.*, 2008).

The crystal packing features C—H···O hydrogen bonds. Atom C11 at *x, y, z* donates one proton to atom O1 at *1 - x, -1/2 + y, 3/2 - z*, forming C(6) zigzag chains along the *b* axis (Fig. 2). The crystal packing also features three weak C—H···π interactions, the first one between a benzene H9 atom and the thiazole ring (S1/N1/C1/C2/C7) of an adjacent molecule, with a C9—H9···*Cg*1^ii^ seperation of 2.92 Å, the second one between a benzene H10 atom and the benzene ring (C2–C7) of a neighbouring molecule, with a C10—H10···*Cg*2^ii^ seperation of 2.90 Å and the third one between a benzene H3 atom and the benzene ring (C8–C13) of a neighbouring molecule, with a C3—H3···*Cg*3^iii^ seperation of 2.99 Å (Table 1 and Fig. 3; *Cg*1, *Cg*2 and *Cg*3 are the centroids of the (S1/N1/C1/C2/C7) thiazole ring, (C2–C7) benzene ring and (C8–C13) benzene ring, respectively. symmetry code as in Fig. 3).

### Experimental

A mixture of 2-nitrobenzaldehyde (1 g, 6.6 mmol), 2-aminobenzenethiol (0.827 g, 6.6 mmol) and bakers' yeast (2.05 g) were stirred at room temperature for 24 h in dichloro methane(DCM). After completion of the reaction, the bakers' yeast was filtered through a bed of Celite, and the filtrate was concentrated under reduced pressure. On cooling, the solid product (1.60 g, 94%) obtained was separated and crystallized from ethylacetate to afford the title compound as yellow blocks.

### Refinement

All the H atoms were positioned geometrically, with C–H = 0.93–0.96 Å and constrained to ride on their parent atom, with *U*_iso_(H) = 1.2*U*_eq_(C).

### Crystal data

**Table:**

C_13_H_8_N_2_O_2_S	*F*(000) = 528
*M**_r_* = 256.27	*D*_x_ = 1.459 Mg m^−^^3^
Monoclinic, *P*2_1_/*c*	Mo *K*α radiation, λ = 0.71073 Å
Hall symbol: -P 2ybc	Cell parameters from 3261 reflections
*a* = 7.6092 (2) Å	θ = 2.3–29.5°
*b* = 12.7854 (3) Å	µ = 0.27 mm^−^^1^
*c* = 11.9938 (3) Å	*T* = 293 K
β = 90.556 (2)°	Block, yellow
*V* = 1166.78 (5) Å^3^	0.24 × 0.22 × 0.16 mm
*Z* = 4	

### Data collection

**Table:**

Bruker APEXII CCD diffractometer	3258 independent reflections
Radiation source: fine-focus sealed tube	2559 reflections with *I* > 2σ(*I*)
Graphite monochromator	*R*_int_ = 0.027
Detector resolution: 10.0 pixels mm^-1^	θ_max_ = 29.5°, θ_min_ = 2.3°
ω scans	*h* = −6→10
Absorption correction: multi-scan (*SADABS*; Sheldrick, 1996)	*k* = −17→17
*T*_min_ = 0.937, *T*_max_ = 0.958	*l* = −16→16
14037 measured reflections	

### Refinement

**Table:**

Refinement on *F*^2^	Primary atom site location: structure-invariant direct methods
Least-squares matrix: full	Secondary atom site location: difference Fourier map
*R*[*F*^2^ > 2σ(*F*^2^)] = 0.039	Hydrogen site location: inferred from neighbouring sites
*wR*(*F*^2^) = 0.113	H-atom parameters constrained
*S* = 1.05	*w* = 1/[σ^2^(*F*_o_^2^) + (0.0565*P*)^2^ + 0.2357*P*] where *P* = (*F*_o_^2^ + 2*F*_c_^2^)/3
3258 reflections	(Δ/σ)_max_ < 0.001
163 parameters	Δρ_max_ = 0.24 e Å^−^^3^
0 restraints	Δρ_min_ = −0.32 e Å^−^^3^

### Special details

**Table:**

Geometry. All e.s.d.'s (except the e.s.d. in the dihedral angle between two l.s. planes) are estimated using the full covariance matrix. The cell e.s.d.'s are taken into account individually in the estimation of e.s.d.'s in distances, angles and torsion angles; correlations between e.s.d.'s in cell parameters are only used when they are defined by crystal symmetry. An approximate (isotropic) treatment of cell e.s.d.'s is used for estimating e.s.d.'s involving l.s. planes.
Refinement. Refinement of *F*^2^ against ALL reflections. The weighted *R*-factor *wR* and goodness of fit *S* are based on *F*^2^, conventional *R*-factors *R* are based on *F*, with *F* set to zero for negative *F*^2^. The threshold expression of *F*^2^ > σ(*F*^2^) is used only for calculating *R*-factors(gt) *etc*. and is not relevant to the choice of reflections for refinement. *R*-factors based on *F*^2^ are statistically about twice as large as those based on *F*, and *R*- factors based on ALL data will be even larger.

### Fractional atomic coordinates and isotropic or equivalent isotropic displacement parameters (Å^2^)

**Table:**

	*x*	*y*	*z*	*U*_iso_*/*U*_eq_	
C1	0.23422 (17)	0.05259 (10)	0.59828 (11)	0.0353 (3)	
C2	0.16130 (18)	0.21138 (10)	0.64971 (11)	0.0363 (3)	
C3	0.0966 (2)	0.29175 (12)	0.71628 (13)	0.0480 (3)	
H3	0.0530	0.2776	0.7869	0.058*	
C4	0.0984 (2)	0.39208 (12)	0.67587 (16)	0.0548 (4)	
H4	0.0561	0.4462	0.7199	0.066*	
C5	0.1620 (2)	0.41411 (12)	0.57096 (16)	0.0560 (4)	
H5	0.1621	0.4830	0.5460	0.067*	
C6	0.2249 (2)	0.33699 (12)	0.50273 (15)	0.0531 (4)	
H6	0.2667	0.3523	0.4320	0.064*	
C7	0.22399 (19)	0.23476 (11)	0.54327 (12)	0.0404 (3)	
C8	0.26409 (17)	−0.06135 (10)	0.60273 (11)	0.0362 (3)	
C9	0.2087 (2)	−0.12556 (11)	0.51565 (13)	0.0465 (3)	
H9	0.1533	−0.0963	0.4536	0.056*	
C10	0.2349 (2)	−0.23238 (12)	0.52027 (15)	0.0542 (4)	
H10	0.1979	−0.2743	0.4612	0.065*	
C11	0.3152 (2)	−0.27701 (11)	0.61136 (16)	0.0546 (4)	
H11	0.3325	−0.3490	0.6137	0.065*	
C12	0.3705 (2)	−0.21558 (11)	0.69957 (14)	0.0470 (3)	
H12	0.4235	−0.2455	0.7621	0.056*	
C13	0.34560 (18)	−0.10908 (10)	0.69301 (11)	0.0376 (3)	
N1	0.16909 (16)	0.10658 (8)	0.67886 (9)	0.0385 (3)	
N2	0.42054 (19)	−0.04475 (10)	0.78230 (11)	0.0482 (3)	
O1	0.51720 (18)	0.02623 (10)	0.75526 (12)	0.0675 (4)	
O2	0.3857 (2)	−0.06687 (13)	0.87769 (10)	0.0812 (4)	
S1	0.29145 (6)	0.12103 (3)	0.47927 (3)	0.04852 (13)	

### Atomic displacement parameters (Å^2^)

**Table:**

	*U*^11^	*U*^22^	*U*^33^	*U*^12^	*U*^13^	*U*^23^
C1	0.0351 (6)	0.0335 (6)	0.0372 (6)	0.0036 (5)	−0.0008 (5)	0.0019 (5)
C2	0.0360 (7)	0.0340 (6)	0.0389 (6)	0.0019 (5)	−0.0033 (5)	−0.0014 (5)
C3	0.0543 (9)	0.0430 (7)	0.0469 (8)	0.0053 (6)	0.0005 (7)	−0.0092 (6)
C4	0.0577 (10)	0.0379 (7)	0.0687 (10)	0.0062 (7)	−0.0064 (8)	−0.0153 (7)
C5	0.0586 (10)	0.0316 (7)	0.0776 (11)	−0.0010 (7)	−0.0063 (8)	0.0036 (7)
C6	0.0607 (10)	0.0383 (8)	0.0605 (9)	−0.0017 (7)	0.0062 (8)	0.0109 (7)
C7	0.0416 (7)	0.0338 (6)	0.0459 (7)	0.0012 (5)	0.0020 (6)	0.0019 (5)
C8	0.0339 (6)	0.0326 (6)	0.0422 (7)	0.0034 (5)	−0.0006 (5)	−0.0012 (5)
C9	0.0464 (8)	0.0423 (7)	0.0506 (8)	0.0074 (6)	−0.0117 (7)	−0.0064 (6)
C10	0.0537 (9)	0.0404 (8)	0.0681 (11)	0.0038 (7)	−0.0123 (8)	−0.0167 (7)
C11	0.0561 (9)	0.0309 (7)	0.0766 (11)	0.0033 (6)	−0.0052 (8)	−0.0038 (7)
C12	0.0489 (8)	0.0362 (7)	0.0559 (8)	0.0048 (6)	−0.0032 (7)	0.0064 (6)
C13	0.0373 (7)	0.0336 (6)	0.0417 (7)	0.0013 (5)	−0.0006 (5)	−0.0005 (5)
N1	0.0442 (6)	0.0345 (5)	0.0368 (5)	0.0048 (4)	0.0009 (5)	0.0007 (4)
N2	0.0552 (8)	0.0416 (6)	0.0476 (7)	0.0074 (6)	−0.0130 (6)	−0.0025 (5)
O1	0.0702 (8)	0.0496 (7)	0.0822 (9)	−0.0119 (6)	−0.0263 (7)	−0.0049 (6)
O2	0.1183 (13)	0.0836 (10)	0.0414 (6)	0.0026 (9)	−0.0075 (7)	−0.0013 (6)
S1	0.0620 (3)	0.0411 (2)	0.0428 (2)	0.00900 (16)	0.01624 (17)	0.00421 (14)

### Geometric parameters (Å, º)

**Table:**

C1—N1	1.2906 (17)	C7—S1	1.7247 (14)
C1—C8	1.4752 (18)	C8—C13	1.3844 (19)
C1—S1	1.7335 (13)	C8—C9	1.391 (2)
C2—N1	1.3858 (17)	C9—C10	1.381 (2)
C2—C3	1.3940 (19)	C9—H9	0.9300
C2—C7	1.400 (2)	C10—C11	1.371 (2)
C3—C4	1.371 (2)	C10—H10	0.9300
C3—H3	0.9300	C11—C12	1.380 (2)
C4—C5	1.381 (3)	C11—H11	0.9300
C4—H4	0.9300	C12—C13	1.3770 (18)
C5—C6	1.371 (3)	C12—H12	0.9300
C5—H5	0.9300	C13—N2	1.4617 (18)
C6—C7	1.395 (2)	N2—O2	1.2103 (18)
C6—H6	0.9300	N2—O1	1.2142 (19)
			
N1—C1—C8	124.15 (12)	C13—C8—C1	122.09 (12)
N1—C1—S1	116.61 (10)	C9—C8—C1	120.68 (12)
C8—C1—S1	119.24 (10)	C10—C9—C8	120.72 (14)
N1—C2—C3	125.64 (13)	C10—C9—H9	119.6
N1—C2—C7	115.00 (11)	C8—C9—H9	119.6
C3—C2—C7	119.36 (13)	C11—C10—C9	120.44 (15)
C4—C3—C2	118.84 (15)	C11—C10—H10	119.8
C4—C3—H3	120.6	C9—C10—H10	119.8
C2—C3—H3	120.6	C10—C11—C12	120.29 (14)
C3—C4—C5	121.16 (15)	C10—C11—H11	119.9
C3—C4—H4	119.4	C12—C11—H11	119.9
C5—C4—H4	119.4	C13—C12—C11	118.56 (14)
C6—C5—C4	121.63 (15)	C13—C12—H12	120.7
C6—C5—H5	119.2	C11—C12—H12	120.7
C4—C5—H5	119.2	C12—C13—C8	122.75 (13)
C5—C6—C7	117.59 (16)	C12—C13—N2	117.52 (13)
C5—C6—H6	121.2	C8—C13—N2	119.58 (11)
C7—C6—H6	121.2	C1—N1—C2	110.13 (11)
C6—C7—C2	121.41 (14)	O2—N2—O1	124.46 (15)
C6—C7—S1	129.23 (12)	O2—N2—C13	118.28 (14)
C2—C7—S1	109.36 (10)	O1—N2—C13	117.25 (13)
C13—C8—C9	117.22 (12)	C7—S1—C1	88.91 (6)
			
N1—C2—C3—C4	179.67 (15)	C10—C11—C12—C13	−1.0 (3)
C7—C2—C3—C4	−0.8 (2)	C11—C12—C13—C8	1.4 (2)
C2—C3—C4—C5	0.3 (3)	C11—C12—C13—N2	−174.13 (15)
C3—C4—C5—C6	0.4 (3)	C9—C8—C13—C12	−0.9 (2)
C4—C5—C6—C7	−0.5 (3)	C1—C8—C13—C12	178.36 (14)
C5—C6—C7—C2	0.0 (2)	C9—C8—C13—N2	174.55 (14)
C5—C6—C7—S1	179.49 (13)	C1—C8—C13—N2	−6.2 (2)
N1—C2—C7—C6	−179.77 (14)	C8—C1—N1—C2	178.70 (12)
C3—C2—C7—C6	0.7 (2)	S1—C1—N1—C2	−0.41 (15)
N1—C2—C7—S1	0.64 (16)	C3—C2—N1—C1	179.35 (14)
C3—C2—C7—S1	−178.90 (11)	C7—C2—N1—C1	−0.16 (17)
N1—C1—C8—C13	−47.3 (2)	C12—C13—N2—O2	−52.8 (2)
S1—C1—C8—C13	131.81 (12)	C8—C13—N2—O2	131.55 (16)
N1—C1—C8—C9	131.96 (15)	C12—C13—N2—O1	125.64 (16)
S1—C1—C8—C9	−48.95 (18)	C8—C13—N2—O1	−50.05 (19)
C13—C8—C9—C10	0.0 (2)	C6—C7—S1—C1	179.76 (16)
C1—C8—C9—C10	−179.32 (14)	C2—C7—S1—C1	−0.69 (11)
C8—C9—C10—C11	0.4 (3)	N1—C1—S1—C7	0.67 (12)
C9—C10—C11—C12	0.1 (3)	C8—C1—S1—C7	−178.49 (11)

### Hydrogen-bond geometry (Å, º)

**Table:**

*D*—H···*A*	*D*—H	H···*A*	*D*···*A*	*D*—H···*A*
C11—H11···O1^i^	0.93	2.51	3.236 (2)	135
C9—H9···*Cg*1^ii^	0.93	2.92	3.468 (2)	119
C10—H10···*Cg*2^ii^	0.93	2.90	3.536 (2)	127
C3—H3···*Cg*3^iii^	0.93	2.99	3.673 (2)	132

Symmetry codes: (i) −*x*+1, *y*−1/2, −*z*+3/2; (ii) −*x*, −*y*, −*z*+1; (iii) −*x*, *y*+1/2, −*z*+3/2.
